# Targeting Txnip-mediated metabolic reprogramming has therapeutic potential for osteoarthritis

**DOI:** 10.1038/s41420-025-02394-z

**Published:** 2025-03-20

**Authors:** Xiankun Cao, Xiao Yang, Pu Zhang, Jianguang Xu, Jie Zhao, Erzhu Yang

**Affiliations:** 1 Shanghai Key Laboratory of Orthopedic Implants, Department of Orthopedic Surgery, Shanghai Ninth People’s Hospital, Shanghai Jiao Tong University School of Medicine, Shanghai, 200011 PR China; 2 Department of Orthopedics Surgery, Shanghai Sixth People’s Hospital, Shanghai Jiao Tong University School of Medicine, Shanghai, 200011 PR China

**Keywords:** Mechanisms of disease, Glycobiology

## Abstract

Osteoarthritis (OA) inflammatory microenvironment triggered glucose metabolism and mitochondrial dysfunction in chondrocytes, leading to a shift of metabolic tendency between oxidative phosphorylation and anaerobic glycolysis. Thioredoxin-interacting protein (Txnip) increased production of reactive oxygen species (ROS), which exacerbates oxidative stress, inflammation and further accelerates cartilage degeneration and extracellular matrix (ECM) degradation. Txnip expression is also positively correlated with several critical pathological glucose and lipid metabolism processes beyond inflammation and endoplasmic reticulum stress (ERS). While the role of Txnip-mediated chondrocyte metabolic reprogramming in OA has not been explored. This study focuses on the unexplored role of Txnip-mediated chondrocyte metabolic reprogramming in chondrogenesis and ECM deposition. The study reveals that upregulated glycolysis after Txnip knockdown significantly contributes to mouse chondrogenesis and ECM deposition. Moreover, verapamil, a clinically used drug that targets Txnip, shows potential for treating mouse OA. These findings suggest that targeting Txnip-mediated metabolic reprogramming could offer a novel therapeutic strategy for OA treatment.

## Introduction

Osteoarthritis (OA) is a degenerative joint disorder characterized by the progressive destruction of articular cartilage, subchondral bone remodeling, and synovial inflammation, leading to chronic pain and disability [1, 2]. Chondrocytes, the sole cellular inhabitants of articular cartilage, play a crucial role in delicate cartilage homeostasis by balancing anabolic and catabolic processes [3]. The inflammatory microenvironment in OA triggers mitochondrial dysfunction in chondrocytes, culminating in a complicated metabolic disturbance. The dynamic shift from anaerobic glycolysis to transient oxidative phosphorylation and finally back to anaerobic glycolysis happened to adapt these microenvironmental changes [4]. This metabolic reprogramming finally precipitates an increased production of reactive oxygen species (ROS), leading to oxidative stress that exacerbates the inflammatory milieu [5–8]. The interplay between inflammation and oxidative stress, driven by these metabolic alterations, accelerates cartilage degeneration and ECM degradation [6].

Exploring the crucial metabolic regulatory factors as therapeutic targets in OA research field has been a hot issue during the last decade. Recently, the finding of c-Fos controlled early metabolic reprogramming from anaerobic glycolysis to transient oxidative phosphorylation drives DCA (dichloroacetic acid, inhibitor of Pdk) to become a potential early OA therapeutic approach [9]. The novel compound FPH2 promotes the accumulation of beneficial metabolites and energy production by inhibiting carnitine palmityl transferase I (CPT1)-mediated fatty acid oxidation and altering glucose and amino acid metabolism, which provide chondrocytes with a more suitable metabolic environment for their survival and functional maintenance [10]. All similar research findings mean huge translational potential for identifying metabolic regulatory factors.

Thioredoxin-interacting protein (TXNIP) functions predominantly as a crucial physiological inhibitor of intracellular thioredoxin (TXN) and together with an array of coenzymes forms an essential component of the cellular TXNIP-TXN redox system, significantly influencing redox reactions under various pathological conditions [11, 12]. Therefore, the role and mechanism of TXNIP-mediated oxidative stress and subsequent inflammation activation on OA development have been widely investigated [13–19]. Meanwhile, recent studies have established a positive correlation between TXNIP expression levels and several critical pathological glucose and lipid metabolism processes beyond inflammation and ERS [12, 20–23]. However, the correlation between TXNIP expression and OA development is still controversial, and the role of TXNIP-mediated chondrocyte metabolic reprogramming has not been explored up to now.

This study focuses on the unrevealed role of Txnip-mediated chondrocyte metabolic reprogramming on chondrogenesis and extracellular matrix (ECM) deposition. We elucidate that the upregulated glycolysis after Txnip knockdown contributes a lot to chondrogenesis and ECM deposition. The clinically used drug verapamil has the potential for OA treatment by targeting Txnip.

## Results

### TXNIP expression upregulation during the development of OA

**Fig. 1:**
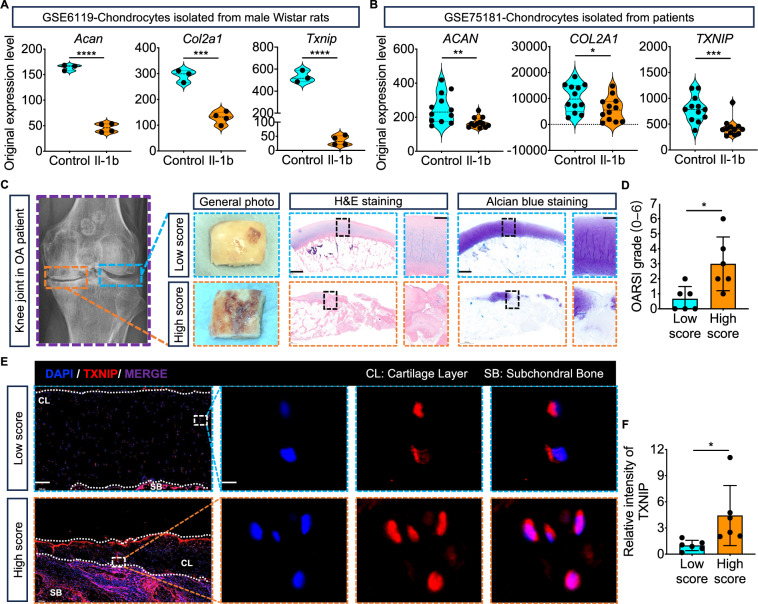
TXNIP expression upregulation during the development of OA. **A** Original expression of *Acan*, *Col2a1*, *Txnip* from rat chondrocytes treated with or without Il-1b. (GSE6119: Control group *n* = 3; Il-1b group *n* = 4). **B** Original expression of *ACAN*, *COL2A1*, *TXNIP* from human chondrocytes treated with or without IL-1b. (GSE75181: Control group *n* = 12; IL-1b group *n* = 12). **C** Representative images of X-ray, H&E, and Alcian blue staining of the high score and low score sides of human OA knee articular. Scale bar = 2000 um, and 500 um. **D** OARSI grade scoring of the high score and low score sides of human OA knee articular. (*n* = 6 per group, 1 technical replicate of 6 biological replicates). **E** Representative TXNIP immunofluorescence images of the high score and low score sides of human OA knee articular. Scale bar = 200 um, and 5 um. **F** Quantitative of TXNIP intensity of the high score and low score sides of human OA knee articular. (*n* = 6 per group, 1 technical replicate of 6 biological replicates). Data are expressed as mean $\pm$ SD (ns, no significance; *p < 0.05; **p < 0.01; ***p < 0.001; ****p < 0.0001 by Student’s *t*-test (**A**, **B**, **D**, **F**)).

Although datasets of both rats and patients (GSE6119: chondrocytes isolated from male Wistar rats; GSE75181: chondrocytes isolated from patients) show downregulated *TXNIP* mRNA levels in IL-1$\beta$ stimulated chondrocytes compared with the control group (Fig. 1A, B). The H&E, Alcian blue, and immunofluorescence staining (IF) observed dramatical upregulation of TXNIP protein expression in the high score side of human OA knee articular compared with the low score side (Fig. 1C–F).

### Txnip expression is negative correlation with chondrogenesis and ECM deposition in vitro

**Fig. 2:**
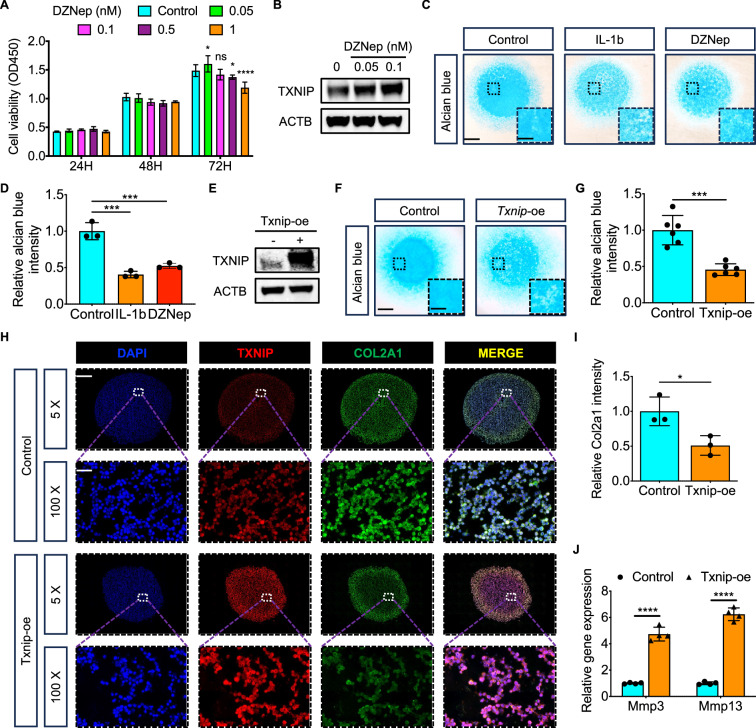
Upregulated Txnip expression inhibits chondrogenesis and ECM deposition in vitro. **A** Cell viability of chondrocytes treated with control, 0.05, 0.1, 0.5, and 1nM DZNep for 24, 48, 72 h. (*n* = 3 per group, 1 technical replicate of 3 biological replicates). **B** Txnip protein expression in chondrocytes treated with control, 0.05, and 0.1nM DZNep. (*n* = 3 per group, 1 technical replicate of 3 biological replicates). **C** Representative alcian blue staining images of chondrocytes treated with control, Il-1b, or 0.1 nM DZNep under high-density culturing in chondrogenesis medium for 9 days. Scale bar = 1.2 mm, and 0.4 mm. **D** Quantification of alcian blue intensity of chondrocytes treated with control, Il-1b, or 0.1 nM DZNep under high-density culturing in chondrogenesis medium for 9 days. (*n* = 3 per group, 1 technical replicate of 3 biological replicates). **E** Txnip protein expression in ATDC5 treated with or without Txnip-overexpression lentivirus. (*n* = 3 per group, 1 technical replicate of 3 biological replicates). **F** Representative alcian blue staining images of ATDC5 treated with or without Txnip-overexpression lentivirus under high-density culturing in chondrogenesis medium for 9 days. Scale bar = 1.2 mm, and 0.4 mm. **G** Quantification of alcian blue intensity of chondrocytes treated with or without Txnip-overexpression lentivirus after high-density culturing in chondrogenesis medium for 7 days. (*n* = 6 per group, 1 technical replicate of 6 biological replicates). **H** Representative Txnip, Col2a1 immunofluorescence images of ATDC5 treated with or without Txnip-overexpression lentivirus under pellet culturing in chondrogenesis medium for 21 days. Scale bar = 600 um, and 30 um. **I** Quantification of Col2a1 intensity of ATDC5 treated with or without Txnip-overexpression lentivirus under pellet culturing in chondrogenesis medium for 21 days. (*n* = 3 per group, 1 technical replicate of 3 biological replicates). **J** Relative *Mmp3*, *Mmp9* mRNA levels of ATDC5 treated with or without Txnip-overexpression lentivirus in chondrogenesis medium for 9 days. (*n* = 4 per group, 1 technical replicate of 4 biological replicates). Data are expressed as mean $\pm$ SD (ns, no significance; *p < 0.05; **p < 0.01; ***p < 0.001; ****p < 0.0001 by Student’s *t*-test (**G**, **I**, **J**) or One-way ANOVA (**A**, **D**)).

The safe dose of DZNep under 0.1nM was demonstrated by the CCK-8 experiment (Fig. 2A). After identifying the inductive effect of DZNep on Txnip (Fig. 2B), the chondrogenesis and ECM deposition change after DZNep treatment was explored. A similar harmful role of DZNep treatment with Il-1$\beta$ treatment was observed by Alcian staining compared with the control group (Fig. 2C, D). Specific Txnip overexpression was also conducted (Fig. 2E) and significant inhibition role of Txnip overexpression with DZNep treatment on chondrogenesis and ECM deposition was observed by Alcian staining (Fig. 2F, G). Moreover, further pellet culturing and IF staining displayed decreased Col2a1 expression after Txnip overexpression, which was suited with the significant upregulated *Mmp3*, *Mmp13* levels in Txnip overexpression chondrocytes (Fig. 2H–J).

**Fig. 3:**
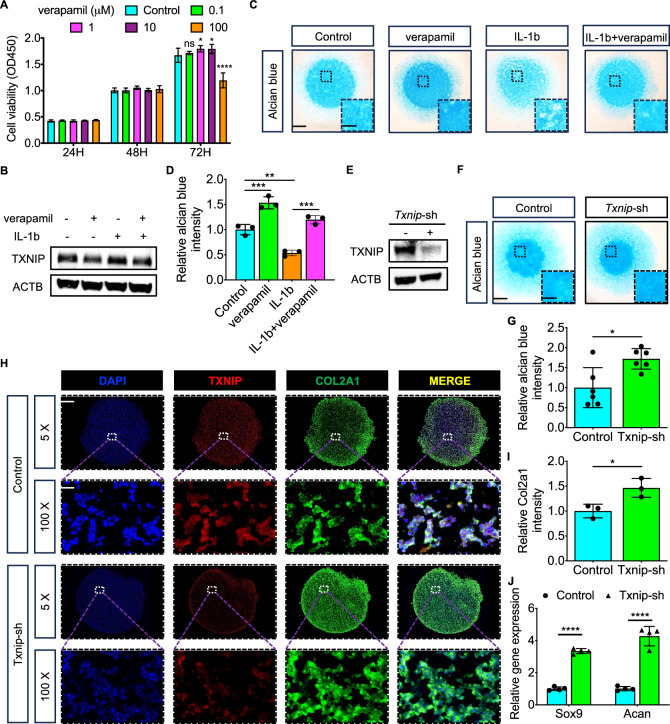
Downregulated Txnip expression promotes chondrogenesis and ECM deposition in vitro. **A** Cell viability of chondrocytes treated with control, 0.1, 1, 10, and 100 uM verapamil for 24, 48, 72 h. (*n* = 3 per group, 1 technical replicate of 3 biological replicates). **B** Txnip protein expression in chondrocytes treated with or without 10 uM verapamil under normal or Il-1b treatment. (*n* = 3 per group, 1 technical replicate of 3 biological replicates). **C** Representative alcian blue staining images of chondrocytes treated with control, Il-1b, verapamil, or Il-1b and verapamil under high-density culturing in chondrogenesis medium for 9 days. Scale bar = 1.2 mm, and 0.4 mm. **D** Quantification of alcian blue intensity of chondrocytes treated with control, Il-1b, verapamil, or Il-1b and verapamil under high-density culturing in chondrogenesis medium for 9 days. (*n* = 3 per group, 1 technical replicate of 3 biological replicates). **E** Txnip protein expression in chondrocytes treated with or without Txnip-silence lentivirus. (*n* = 3 per group, 1 technical replicate of 3 biological replicates). **F** Representative alcian blue staining images of ATDC5 treated with or without Txnip-silence lentivirus under high-density culturing in chondrogenesis medium for 9 days. Scale bar = 1.2 mm, and 0.4 mm. **G** Quantification of alcian blue intensity of ATDC5 treated with or without Txnip-silence lentivirus under high-density culturing in chondrogenesis medium for 9 days. (*n* = 6 per group, 1 technical replicate of 6 biological replicates). **H** Representative Txnip, Col2a1 immunofluorescence images of ATDC5 treated with or without Txnip-silence lentivirus under pellet culturing in chondrogenesis medium for 21 days. Scale bar = 600 um, and 30 um. **I** Quantification of Col2a1 intensity of ATDC5 treated with or without Txnip-silence lentivirus under pellet culturing in chondrogenesis medium for 21 days. (*n* = 3 per group, 1 technical replicate of 3 biological replicates). **J** Relative *Sox9*, *Acan* mRNA levels of ATDC5 treated with or without Txnip-silence lentivirus in chondrogenesis medium for 9 days. (*n* = 4 per group, 1 technical replicate of 4 biological replicates). Data are expressed as mean $\pm$ SD (ns, no significance; *p < 0.05; **p < 0.01; ***p < 0.001; ****p < 0.0001 by Student’s *t*-test (**G**, **I**, **J**) or One-way ANOVA (**A**, **D**)).

The safe dose of verapamil under 10 uM was demonstrated by the CCK-8 experiment (Fig. 3A). After identifying the inhibiting effect of verapamil on Txnip both in physiological and pathological conditions (Fig. 3B), the chondrogenesis and ECM deposition change after verapamil treatment was explored. A promotive role of verapamil treatment compared with the control group and the rescue effect of verapamil under Il-1$\beta$ treatment were both observed by Alcian staining (Fig. 3C, D). Specific Txnip silence was also conducted (Fig. 3E) and consistent promotion effect of Txnip silence with verapmail treatment on chondrogenesis and ECM deposition was observed by Alcian staining (Fig. 3F, G). Moreover, further pellet culturing and IF staining displayed increased Col2a1 expression after Txnip silence, which was suited with the significant upregulated *Sox9*, *Acan* levels in Txnip silence chondrocytes (Fig. 3H–J).

### Txnip expression is harmful to chondrocyte proliferation and promotes apoptosis

**Fig. 4:**
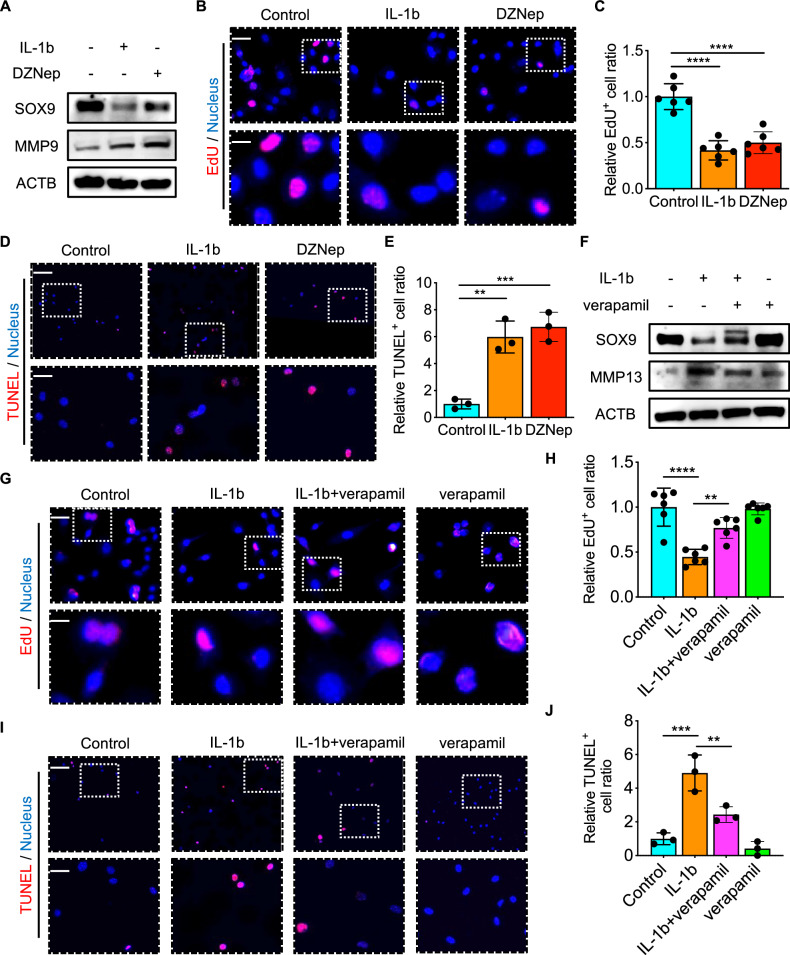
Txnip expression is harmful to chondrocyte proliferation and promotes apoptosis. **A** Sox9, MMP9 protein expression in chondrocytes treated with control, Il-1b, 0.1nM DZNep for 24 h. **B** Representative EdU immunofluorescence images of chondrocytes treated with control, Il-1b, 0.1nM DZNep for 24 h. Scale bar = 15 um, and 5 um. **C** Quantification of EdU^+^ chondrocytes treated with control, Il-1b, 0.1nM DZNep for 24 h. (*n* = 6 per group, 1 technical replicate of 6 biological replicates). **D** Representative TUNEL immunofluorescence images of chondrocytes treated with control, Il-1b, 0.1nM DZNep for 24 h. Scale bar = 100 um, and 25 um. **E** Quantification of TUNEL ^+^ chondrocytes treated with control, Il-1b, 0.1nM DZNep for 24 h. (*n* = 3 per group, 1 technical replicate of 3 biological replicates). **F** Sox9, MMP13 protein expression in chondrocytes treated with control, Il-1b, verapamil, or Il-1b and verapamil for 24 h. **G** Representative EdU immunofluorescence images of chondrocytes treated with control, Il-1b, verapamil, or Il-1b and verapamil for 24 h. Scale bar = 15 um, and 5 um. **H** Quantification of EdU^+^ chondrocytes treated with control, Il-1b, verapamil, or Il-1b and verapamil for 24 h. (*n* = 6 per group, 1 technical replicate of 6 biological replicates). **I** Representative TUNEL immunofluorescence images of chondrocytes treated with control, Il-1b, verapamil, or Il-1b and verapamil for 24 h. Scale bar = 100 um, and 25 um. **J** Quantification of TUNEL ^+^ chondrocytes treated with control, Il-1b, verapamil, or Il-1b and verapamil for 24 h. (*n* = 3 per group, 1 technical replicate of 3 biological replicates). Data are expressed as mean $\pm$ SD (ns, no significance; *p < 0.05; **p < 0.01; ***p < 0.001; ****p < 0.0001 by One-way ANOVA (**C**, **E**, **H**, **J**)).

The downregulation of SOX9 expression and upregulation of MMP9 expression were observed in the DZNep group and Il-1$\beta$ group, compared with the control group (Fig. 4A). The chondrocyte proliferation impartation happened both under Il-1$\beta$ and DZNep-induced Txnip high expression conditions and the apoptosis increased at the same time (Fig. 4B–E). Consistent with our previous experiment results, verapamil treatment rescues SOX9 and inhibits MMP13 expression under Il-1$\beta$ stimulation (Fig. 4F). The declined chondrocyte proliferation and promoted apoptosis were also recovered after verapamil treatment under Il-1$\beta$ stimulation (Fig. 4G–J).

### Downregulated Txnip expression influences glucose utilized efficiency and promotes glycolysis metabolic reprogramming

**Fig. 5:**
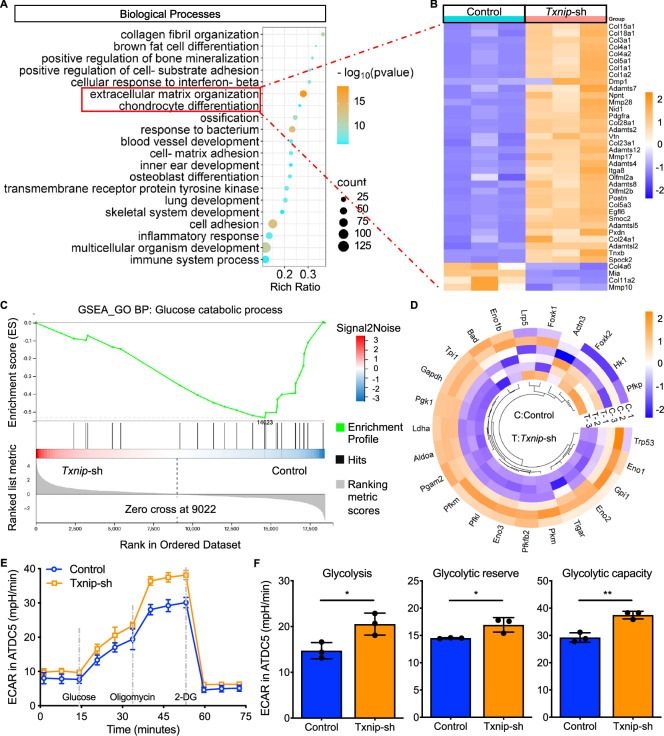
Downregulated Txnip expression influences glucose utilized efficiency and promotes glycolysis metabolic reprogramming. **A** GO enrichment analysis of negative control and *Txnip*-silenced ATDC5 treated with 7 days chondrogenesis induction. (*n* = 3 per group, 1 technical replicate of 3 biological replicates). **B** Heatmap displayed the expression change of genes related to extracellular matrix organization and chondrocyte differentiation. (*n* = 3 per group, 1 technical replicate of 3 biological replicates). **C** Glucose catabolic process by GSEA analysis of negative control and *Txnip*-silenced ATDC5 treated with 7 days chondrogenesis induction. (*n* = 3 per group, 1 technical replicate of 3 biological replicates). **D** Heatmap displayed the expression change of genes related to glucose catabolic process. (*n* = 3 per group, 1 technical replicate of 3 biological replicates). **E** EACR of negative control and *Txnip*-silenced ATDC5 treated with 7 days chondrogenesis induction. **F** Quantification of EACR related glycolysis, glycolytic reserve, and glycolytic capacity. (*n* = 3 per group, 1 technical replicate of 3 biological replicates). Data are expressed as mean $\pm$ SD (ns, no significance; *p < 0.05; **p < 0.01; ***p < 0.001; ****p < 0.001 by Student’s *t*-test (**F**)).

To identify the underlying mechanisms for Txnip-mediated chondrocyte differentiation, The GO enrichment analysis of differential expression genes was conducted and the results showed significant enrichment of biological processes on “extracellular matrix organization” and “chondrocyte differentiation” (Fig. 5A). Lots of genes that promote chondrogenesis were upregulated with the declined Txnip expression (Fig. 5B). Moreover, the Gene Set Enrichment Analysis (GSEA) analysis showed that the glucose catabolic process was downregulated in the *Txnip*-silenced group (Fig. 5C). Most of genes that participate in glucose metabolism were positively correlated with the declined Txnip expression (Fig. 5D). The change of metabolic way after *Txnip*-silence was then detected by seahorse experiments. Significant upregulation of extracellular acidification rate (ECAR) was observed after *Txnip*-silence as shown by the quantification by glycolysis, glycolytic reserve, and glycolytic capacity (Fig. 5E, F).

### Inhibition of *Txnip*-silence induced glycolysis metabolic reprogramming attenuate the ability to promote chondrogenesis

**Fig. 6:**
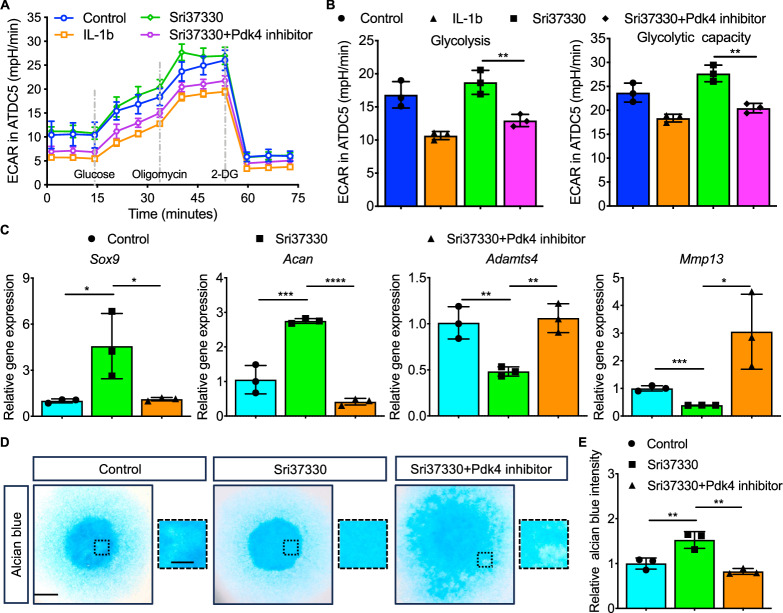
Inhibition of *Txnip*-silence induced glycolysis metabolic reprogramming attenuate the ability to promote chondrogenesis. **A** EACR of ATDC5 treated with negative control, IL-1b, Sri37330, Sri37330+Pdk4 inhibitor. **B** Quantification of EACR related glycolysis and glycolytic capacity. (*n* = 3 per group, 1 technical replicate of 3 biological replicates). **C** Relative *Sox9*, *Acan*, *Adamts4*, and *Mmp13* mRNA levels of ATDC5 treated with or without *Txnip*-silence lentivirus in chondrogenesis medium for 9 days. (*n* = 3 per group, 1 technical replicate of 3 biological replicates). **D** Representative alcian blue staining images of chondrocytes treated with negative control, Sri37330, or Sri37330+Pdk4 inhibitor under high-density culturing in chondrogenesis medium for 9 days. Scale bar = 1.2 mm, and 0.4 mm. **E** Quantification of alcian blue intensity of chondrocytes treated with negative control, Sri37330, or Sri37330+Pdk4 inhibitor under high-density culturing in chondrogenesis medium for 9 days. (*n* = 3 per group, 1 technical replicate of 3 biological replicates). Data are expressed as mean $\pm$ SD (ns, no significance; *p < 0.05; **p < 0.01; ***p < 0.001; ****p < 0.001 by One-way ANOVA (**B**, **C**, **E**)).

Whether *Txnip*-silence induced glycolysis metabolic reprogramming functioned in chondrogenesis was then determined. Compared with the severely impaired glycolysis after IL-1b treatment, Sri37330 (specific Txnip inhibitor) treatment exactly improved the glycolytic capacity and this effect was significantly attenuated by Pdk4 inhibitor (important protein for the transform from oxidative phosphorylation to glycolysis) (Fig. 6A, B). Accompanied by this change, Significant upregulated *Sox9*, *Acan* levels and inhibited *Adamts4*, *Mmp13* levels were also offset (Fig. 6C). A similar phenomenon was observed in the Alician staining reflected the change in ECM deposition (Fig. 6D, E).

### Verapamil has the therapeutic potential for destabilization of medial meniscus (DMM)-induced OA

**Fig. 7:**
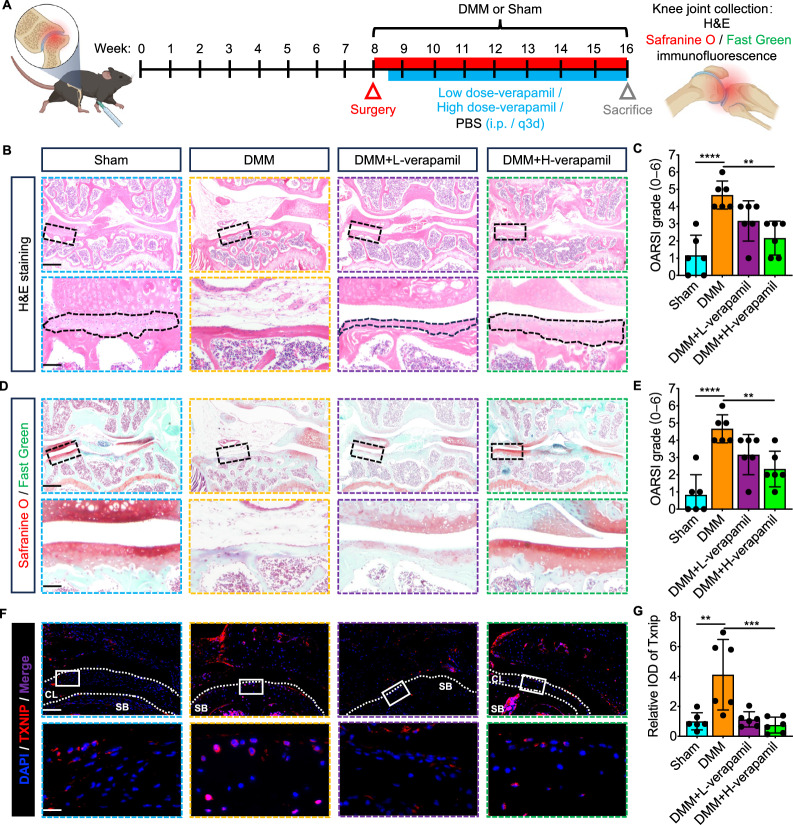
Verapamil has the therapeutic potential for destabilization of DMM-induced OA. **A** Schematic diagram of the therapeutic experiment design and observation indicators of verapamil on DMM-induced OA. **B** Representative H&E staining images of knee joints at a coronal position from mice suffering sham, DMM, DMM with low or high dose verapamil treatment. Scale bar = 100 um, and 15 um. **C** OARSI grade scoring of the knee joints cartilage endplates of (**B**). (*n* = 6 per group, 1 technical replicate of 6 biological replicates). **D** Representative Safranin O-Fast Green staining images of knee joints at a coronal position from mice suffering sham, DMM, DMM with low or high dose verapamil treatment. Scale bar = 100 um, and 15 um. **E** OARSI grade scoring of the knee joints cartilage endplates of (**D**). (*n* = 6 per group, 1 technical replicate of 6 biological replicates). **F** Representative Txnip immunofluorescence images of knee joints at a coronal position from mice suffering sham, DMM, DMM with low or high dose verapamil treatment. Scale bar = 80 um, and 15 um. **G** Quantitative of Txnip intensity of (**F**). (*n* = 6 per group, 1 technical replicate of 6 biological replicates). Data are expressed as mean $\pm$ SD (ns, no significance; *p < 0.05; **p < 0.01; ***p < 0.001; ****p < 0.001 by One-way ANOVA (**C**, **E**, **G**)).

The potential therapeutic possibility of targeting Txnip in DMM-induced OA was then determined through verapamil intraperitoneally injection (Fig. 7A). As shown by the H&E staining, cartilage tissue thickness was significantly restored and the OARIS histological score was decreased after verapamil treatment compared with the DMM group (Fig. 7B, C). The Safranine O-Fast Green staining further showed the degeneration of the cartilage surrounding the tibia and femur was restored and the OARIS histological score was decreased after verapamil treatment compared with the DMM group (Fig. 7D, E). Moreover, IF staining also displayed increased Txnip expression in chondrocytes of DMM-induced mice articulus and the rescue effect of verapamil on pathological Txnip upregulation (Fig. 7F, G).

## Discussion

OA is a chronic inflammatory disease, and inflammatory factors can affect the energy metabolism of chondrocytes. When the energy metabolism of chondrocytes is abnormal, it can decrease the synthesis and degradation of cartilage matrix, and the degradation and destruction of cartilage tissue accelerate the development of OA [24, 25]. The crucial metabolic rewiring from glycolysis to tricarboxylic acid (TCA) cycle-oxidative phosphorylation (OXPHOS) in the early stage [9] and subsequently enhanced glycolysis with mitochondria function disorder induced overmuch ROS generation [26], ATP, NADPH deficient [8] display continuously delicate metabolic regulation variation during this pathological process. Txnip, which was authenticated to be important in glucose transport-related glucolipid metabolism [27–29] and important in chronic inflammatory and metabolic diseases-related glycolysis molecular regulation [23, 30, 31], has been demonstrated to driving oxidative stress, inflammation-induced endoplasmic reticulum stress (ERS), and NLRP3 inflammasome activation [32] through such as Sirtuin 6 (SIRT6) activity decline [14], Peroxisome Proliferators Activated Recepotor δ (PPARδ) activation [15] to caused chondrogenesis and ECM deposition obstruction. However, other papers say that reduced Regulated in development and DNA damage-1 (REDD1)/TXNIP complexes during OA development reduced chondrocyte autophagy through activating mammalian target of rapamycin (mTOR) signaling [13]. The controversial reports of its correlation with OA and the lack of evidence of its role in chondrocyte metabolic reprogramming drove us to conduct this research.

Although mRNA levels of TXNIP were found downregulated in IL-1β stimulated chondrocytes compared to controls in datasets (GSE6119 and GSE75181) from both rats and patients with OA. We observed increased TXNIP protein expression in the high score OA group compared to the low score OA group, supporting the previous research that TXNIP possibly contributes to disease progression. We further demonstrate that TXNIP upregulation by DZNep [33] or lentivirus inhibits chondrogenesis and extracellular matrix (ECM) deposition in vitro, while its downregulation by verapamil [34, 35] or lentivirus promotes these processes. Similar effects were observed in cartilage matrix degradation and synthesis proteins MMP9, MMP13, and SOX9 expression and chondrocyte proliferation and apoptosis behavior. These findings align with the observed exacerbated cartilage degradation and ECM disruption in mice destabilization of the medial meniscus (DMM) model, which partially results from dysregulated increased Txnip. Most importantly, our study also links Txnip to glycolysis metabolic reprogramming, a cellular adaptation to stress conditions often observed in various diseases including cancer and neurodegeneration [36]. In chondrocytes, decreased Txnip expression by Sri37330, a specific Txnip inhibitor [23], correlates with enhanced glycolysis, as evidenced by elevated extracellular acidification rates (ECAR). Moreover, this glycolysis metabolic reprogramming appears to be crucial for Txnip-induced promotion of chondrogenesis, as inhibition of glycolysis by Pdk4 inhibitor [37] attenuates this effect.

The potential therapeutic implications of modulating Txnip activity become evident through verapamil treatment, an established method to decrease Txnip levels [38]. In a mouse model of DMM-induced OA, verapamil treatment significantly ameliorated cartilage degradation and reduced histological scores for OA severity. Notably, verapamil countered the pathological upregulation of Txnip observed in DMM-induced OA, supporting the notion that targeting the TXNIP pathway could mitigate cartilage degeneration in OA.

In conclusion, these experiment data highlight the dual role of Txnip in OA pathogenesis: it promotes cellular oxidative stress, inflammatory responses, and matrix degradation [39] while also inhibiting chondrocyte metabolism towards glycolysis. These insights pave the way for future investigations into Txnip-mediated metabolic reprogramming mechanism and its implications in OA development and therapy. Moreover, drugs, such as verapamil in this paper, targeting the Txnip and abnormal metabolic reprogramming present a novel therapeutic strategy to combat this debilitating condition. However, limitations are still present in this study. Given the complex interplay between inflammation, oxidative stress, metabolic alterations, and cartilage homeostasis in OA, the reason for contradictory Txnip mRNA level and protein expression, and the specific molecular mechanism for Txnip-mediated metabolic reprogramming were not explored in this paper. Meanwhile, therapeutic effect verification of specific Txnip inhibitor Sri37330 in the DMM-induced mouse OA could strengthen the conclusion and benefit the drug development.

## Materials and methods

### Mouse model

The C57BL/6J mice (NO.000013) was purchased from GemPharmatech (Nanjing, China). All animals were ensured under C57BL/6 background and specific-pathogen-free (SPF) grade in this research and were housed in the Department of Laboratory Animal Science at Shanghai Ninth People’s Hospital. All animals were healthy without any diseases and all animal experimental procedures were conducted in strict accordance and approved by the Institutional Animal Care and Experimental Committee of Shanghai Jiao Tong University School of Medicine.

### DMM model and verapamil treatment

Twenty-four 8-week-old male C57BL/6J mice were prepared for the treatment experiment. Eighteen were casually selected and performed destabilization of medial meniscus surgery, which was then divided into three groups randomly for later experiments. DMM group: every 3 days intraperitoneal injections of PBS for 8 weeks (Hyclone, SH30256.01). DMM with Low verapamil group: 8 weeks every 3 days intraperitoneal injections of verapamil (2.11mg/kg; APExBIO, B1867). DMM with High verapamil group: 8 weeks every 3 days intraperitoneal injections of verapamil (4.11mg/kg) [40]. The remaining six mice that underwent the same procedure but without their articular cartilage injury were considered the sham group.

The post-operative infections and pain of operated mice were prevented by gentamicin (i.m., 10 mg/kg) and tramadol (s.c., 25 mg/kg) for the initial first three days. All mice were sacrificed at the designed point, and the surgical lower limbs were collected. The knee articulars were then performed histological experiments. All analyses were described later herein.

### Human cartilage specimen collection and preparation

Human tibial plateau was collected from OA patients undergoing total knee arthroplasty (TKA), and the demographic data of all six patients are detailed in Table 1. We collected these human cartilage samples and divided them into low-score and high-score sides in each sample according to the relative of wear judged and joint clearance height by the OARSI scoring [41]. In the center of each side, we cut an square osteochondral plug and fixed it in 4% paraformaldehyde for further analysis.

**Table 1:** Demographic statistics of patients whose cartilage samples were used in this study.

I.D.	Age	Sex	Kellgren-Lawrence (KL) grade
1	76	M	3
2	62	F	3
3	74	M	4
4	73	F	4
5	71	F	3
6	64	F	4

All patients were from the Chinese Han population and the ethics committee of Shanghai Ninth People’s Hospital approved all the studies involving human articular cartilage (SH9H-2021-T401-4).

### Cell isolation and culture

For mouse primary chondrocytes, three 3-day-old male mice were euthanized and immersed in 75% ethanol for 10 min. Dissect both lower limbs, remove the skin, extract the entire knee joint, and peel off the synovium and muscle tissue. Cut these six cartilage samples into pieces (0.5–1 mm), soak them in a 1% collagenase II solution at 37 °C for 2 h, centrifuge (at 300 × g, 37 °C for 5 min), and resuspend them in complete culture medium (DMEM/F12, containing 5% FBS, 1% penicillin-streptomycin). Primary chondrocytes were cultured in DMEM/F12 (Gibco; Thermo Fisher Scientific, In a limited liability company, DMEM/F2 was supplemented with 5% FBS and 1% penicillin-streptomycin (Gibco, Thermo Fisher Scientific, Co., Ltd.) and 1% insulin transfer iron selenium (ITS) solution, 37 °C, 5% CO_2_.

Mouse ATDC5 immortalized chondrocytes were maintained in DMEM/F12 supplemented with 5% FBS and 1% penicillin-streptomycin (Gibco; Thermo Fisher Scientific, Inc.) at 37 °C with 5% CO_2_.

### Cytotoxic assay

The cytotoxic effects of verapamil, DZNep on primary mouse chondrocytes were determined by the CCK-8 kit (Dojindo Molecular Technology, Japan). Specifically, 100μL medium containing 10% CCK-8 buffer was added to the wells after treatment and incubated in the dark at 37 °C for 2 h. The absorbance was then detected at 450 nm wavelength (650 nm reference) on a microplate reader.

### High-density culture and alcian blue staining

To evaluate chondrogenic differentiation and ECM deposition, 1.5 × 10^5^ primary chondrocytes, ATDC5, Txnip overexpression or Txnip silence ATDC5 were resuspended in 10 µl incomplete MEM/F12 (Gibco; Thermo Fisher Scientific, Inc.) and seeded as micromasses in the bottom of a 24-well plate. The cells were allowed to adhere for 1 h at 37 °C, after which 1 ml MEM/F12 containing 10 ng/ml ITS and 2% FBS was added. After 24 h at 37 °C, the cells were stimulated separately under one of the following conditions: IL-1β (10 ng/ml), verapamil (10 µM) with or without IL-1β, DZNep (0.1 nM), Sri37330, Sri37330 with Pdk4 inhibitor (M77976), and the control groups were cultured in a medium with DMSO only (1:2000) for 9 days at 37 °C. All media were refreshed every other day, and after 9 days the micromasses were stained with alcian blue for 24 h at room temperature (RT).

### Pellet culture

To further assess chondrogenic differentiation and ECM deposition, 1.5 × 10^7^ Negative control, Txnip overexpression, or Txnip silence ATDC5 were pelleted in 15ml centrifuge tubes (200 × g, 37 °C for 5 min) supplemented with specific chondrogenic differentiation medium (Cyagen Biosciences, Inc.) at 37 °C. The media were refreshed every 3 days. After 21 days of culture, the pellets were collected and fixed at RT in 4% paraformaldehyde (PFA) for 5 h and then embedded in an optimal cutting temperature compound (Sakura Finetek USA, Inc.). The samples were then stored at −80 °C overnight and cut to a 20µm thickness using a freezing microtome (Leica Microsystems GmbH) for further experiments.

### EdU staining assay

EdU staining was performed using the EdU poliferation assay kit (Beyotime Biotechnology) according to the manufacturer’s instructions. Briefly, chondrocytes were fixed in 4% paraformaldehyde for 10 min. After washing thrice with PBS, the sections were incubated with the EdU reaction mixture for 2 h at 37 °C in a moist chamber. The nuclei were stained using DAPI. All images were acquired using a DM4000 B epifluorescence microscope (Leica Microsystems GmbH).

### Terminal deoxynucleotidyl transferase deoxyuridine triphosphate nick-end labeling (TUNEL) staining assay

TUNEL staining was performed using the colorimetric TUNEL apoptosis assay kit (Beyotime Biotechnology) according to the manufacturer’s instructions. Briefly, chondrocytes were fixed in 4% paraformaldehyde for 10 min. After washing thrice with PBS, the sections were incubated with the TUNEL reaction mixture for 2 h at 37 °C in a moist chamber. The nuclei were stained using DAPI. All images were acquired using a DM4000 B epifluorescence microscope (Leica Microsystems GmbH).

### RNA-sequencing and analysis

The total RNA from negative control or *Txnip*-silenced ATDC5 cells with 7-day chondrogenesis induction was extracted by TRIzol (Invitrogen, Cat # 15596018). The RNA-seq was then conducted according to the BGISEQ platform’s standard protocol, which including complementary DNA library preparation and sequencing, raw reads, differential gene expression identification, DEGs analysis, GO analysis, KEGG analysis, and GSEA analysis. Differentially expressed genes were clustered by k-means clustering using the Euclidean distance as the distance and the GO plot, heatmap, GSEA plot was reformatted by bioinformatics online website (https://www.bioinformatics.com.cn).

### Seahorse metabolic flux analysis

The ATDC5 cells from negative control or *Txnip*-silence were plated in XF-96 cell culture plates at 1 × 10^4^/well (Seahorse Bioscience, USA) overnight. Then, the cells were treated with negative control, IL-1b, Sri37330, and Sri37330+Pdk4 inhibitor for 24 h. The measurements of extracellular acidification rate (ECAR) [10 mM glucose, 2 *μ*M oligomycin, and 50 mM 2-DG] were carried out according to standard protocols, and the results were detected with the Seahorse XF-96 Flux Analyzer (Seahorse Bioscience), as previously described [42]. Additionally, the glycolysis, glycolytic reserve, glycolytic capacity were calculated by the ECAR changes [43].

### Histology and immunofluorescence (IF) staining

Fixed mouse lower limb and human osteochondral plug samples were embedded in paraffin and subjected to 5 µm thickness histological sectioning. The articular cartilage was stained with hematoxylin and eosin (H&E), alcian blue, and Safranine O/Fast green staining according to manufacturer instructions to evaluate cartilage clefts. The OARSI score was evaluated according to the W. B. van den Berg Standards [41].

For immunofluorescence staining, mouse lower limb, human osteochondral plug, and ATDC5 Pellet histological sectioning were handled in the order of deparaffinization, hydration, antigen retrieval, permeabilization, blocking, and finally incubated with primary antibody (Txnip, Novus Bio, NBP1-54578, 1:100; Col2a1, Abcam, ab34712, 1:100) overnight at 4°C. The Alexa Fluor 555 or 488 conjugate secondary antibodies were then incubated with sections on the next day at room temperature for 1 h. The nuclei were stained in the dark by 4’, 6-diamidino-2-phenylindole (DAPI) at RT for 5 min. After the last PBS wash, The sections were observed and imaged under a DM4000 B epifluorescence microscope (Leica, Germany) and analyzed with ImageJ software.

### Quantitative real-time PCR analysis

To explore the relative gene expression, total RNA was extracted at indicated time points according to the experiment arrangement by the Axygen RNA Miniprep Kit (Axygen, Cat # AP-MN-RNA, Union City, CA, USA). The cDNA was then obtained from the RNA template by using a PrimeScript RT Master Mix kit to reverse transcription (Takara, Cat # RR036A). The primes and samples were prepared according to the instruction of the TBGreen ® Premix Ex Taq ™ kit (Takara, Cat # RR420A) and real-time PCR process (40 cycles: 95 °C for 5 s plus 60 °C for 30 s) was run on ABI 7500 sequencing detection system (Applied Biosystems, Foster City, CA). Specifically, 5 μl TBGreen, 0.2 ul Rox, 0.2 μl forward, 0.2 μl reverse primer, and 3.4 μl ddH2O with 1 μl diluted cDNA were mixed to be the 10 μl total volume liquid for each hole and added into the 384 PCR plate. The specificity of amplification products was judged by melting curve and reverse transcription PCR (RT-PCR). The quantification of each target was normalized to the Actin beta (*Actb*). The primer sequences are listed in Table 2.

**Table 2:** Primer sequences for qPCR.

Gene	Organisms	Forward Sequence (5′-3′)	Reverse Sequence (5′-3′)
Mmp3	Mus musculus	CCCTGCAACCGTGAAGAAGA	GACAGCATCCACCCTTGAGT
Mmp13	Mus musculus	AGAAGTGTGACCCAGCCCTA	GGTCACGGGATGGATGTTCA
Adamts4	Mus musculus	GAGTCCCATTTCCCGCAGA	GCAGGTAGCGCTTTAACCCT
Sox9	Mus musculus	CGTGGACATCGGTGAACTGAG	GGTGCTGCTGATGCCGTAAC
Acan	Mus musculus	GCTACCCTGATCCCTCATCC	GATGTCCTCTTCACCACCCA
Actb	Mus musculus	CCCGCGAGTACAACCTTCT	ATGCCGTGTTCAATGGGGTA

### Western blotting analysis

At designated time points, proteins were extracted using radioimmunoassay (RIPA) lysis buffer (Beyotime, Cat # P0013C, Shanghai, China) plus a mixture of protease and phosphorylase inhibitor cocktail (A+B) (Abmole, Cat # M5293, Cat # M7528). After 13000 x *g* centrifuging for 15 min, the concentration of proteins in the supernatant was detected by Pierce ™ Bicinchoninic Acid (BCA) protein quantitative Kit (Thermo Fisher, Cat # 23225). Then, 5×SDS-sample loading buffer was used to dilute the supernatant protein and heated at 95 degrees for 10 min. After 4–20% SDS-PAGE separation and nitrocellulose filter membrane transferration (GE Healthcare Life Sciences, Pittsburgh, PA, USA), the membrane was soaked in 5% skim milk dissolved in 1 × TBST (Tris-buffered saline with Tween 20) at room temperature for 1 h blocking. The primary antibodies (Txnip, CST, 14715S, 1:1000; Mmp9, Proteintech, 10375-2-AP, 1:1000; Mmp13, Proteintech, 18165-1-AP, 1:1000; Sox9, CST, 82630, 1:1000; ACTB, CST, 8457, 1:1000) were next incubated with membranes at 4 °C overnight. Next day, the secondary fluorescence antibody was incubated in the dark at room temperature for 1 h after 3 times TBST wash. After another 3 times TBST wash, the Odyssey v3.0 image scanning was used to develop the result of reactivity (Li Cor. Inc., Lincoln, NE, USA).

### Statistical analysis

The *n* used in whether mice per group or human specimens indicates the number of biologically independent samples. All the cell experiments and animal sample sizes were predetermined to reach at least 3 biological repeats without prior power calcs and any exclusion criteria. Histological analyses and cell experiment result analyses were done in a blinded fashion and the statistical results were calculated by Prism 8 (GraphPad Software Inc, San Diego, CA, USA). The data were presented mainly in two forms: mean $\pm$ SD, and violin plots with all points. The inter-group variances of most evaluation parameters were similar and the Kolmogorov-Smirnov test was used to determine the normality of the data. For the two groups’ comparisons, significance was analyzed by the 2-tailed, unpaired or paired Student’s *t*-test. When analyzing data among more than two groups, One-way analysis of variance (ANOVA) with turkey’s post-hoc-test was used. Two-way analysis of variance (ANOVA) with sidak’s post hoc test was used. The standard of statistically significant (ns, not significant; *p < 0.05; ** *p* < 0.01; *** *p* < 0.001; **** *p* < 0.0001).

## Supplementary information

Original wb images

## Data Availability

Any information required to reanalyze the data reported in this paper is available from the corresponding author upon request.
