# Supplementary figures and images for: Targeting Txnip-mediated metabolic reprogramming has therapeutic potential for osteoarthritis
Source: Cell Death Discov. 2025 Mar 20;11:110. doi: 10.1038/s41420-025-02394-z (PMC11926230; doi:10.1038/s41420-025-02394-z)

Figure 2B

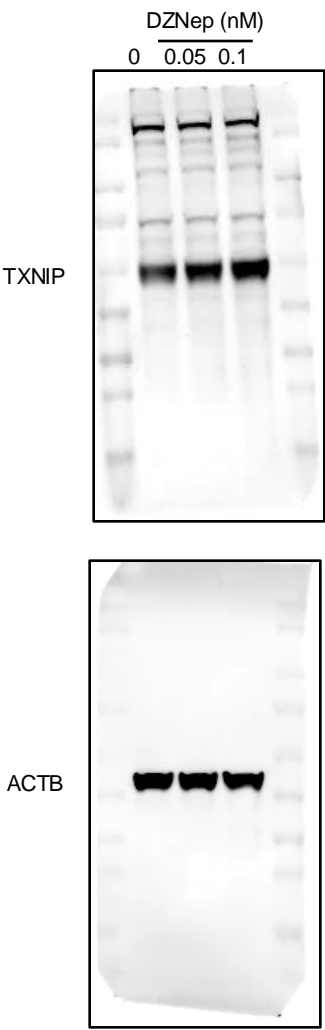

Figure 2E

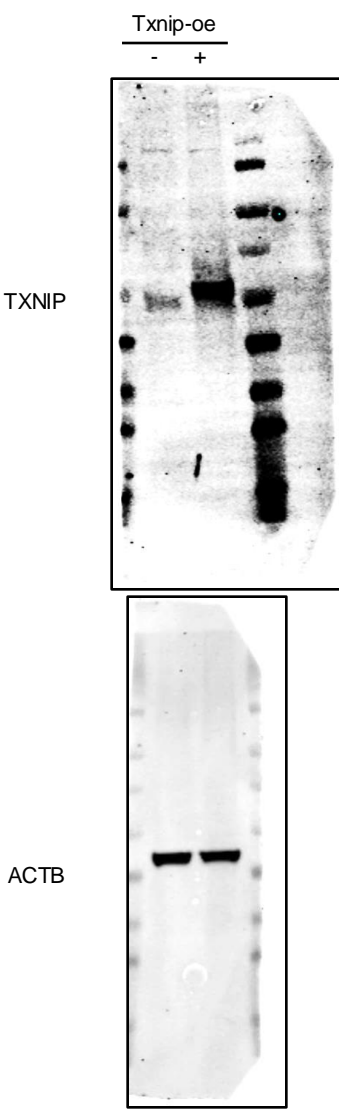

Figure 3B

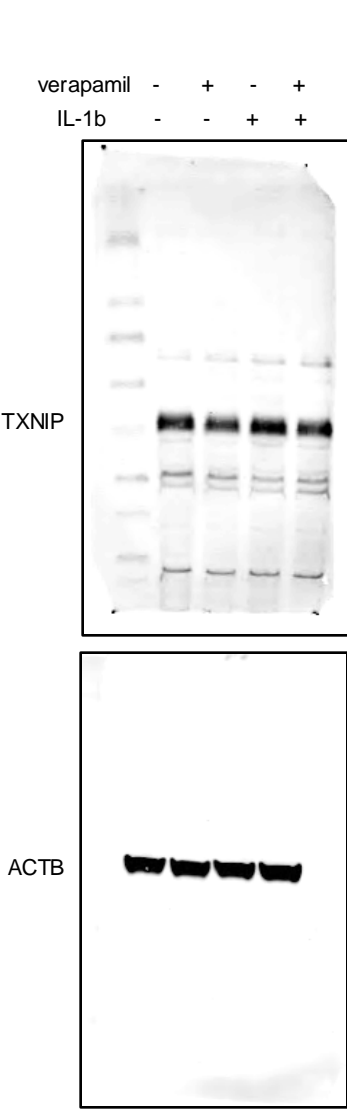

Figure 3E

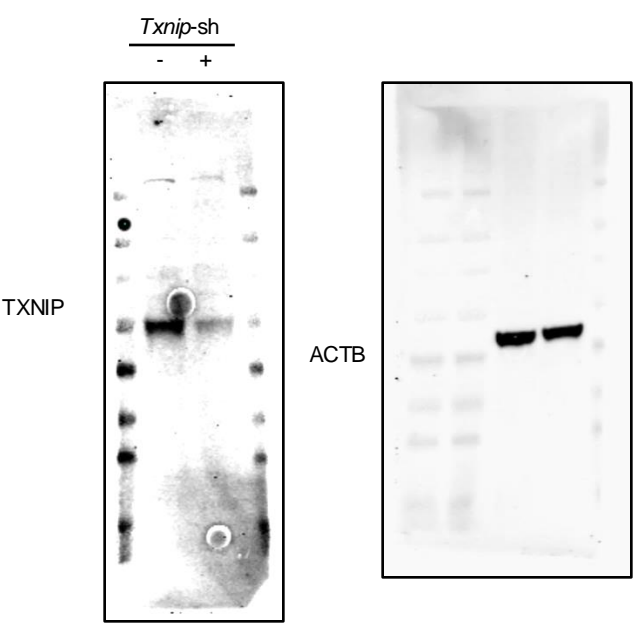

Figure 4A

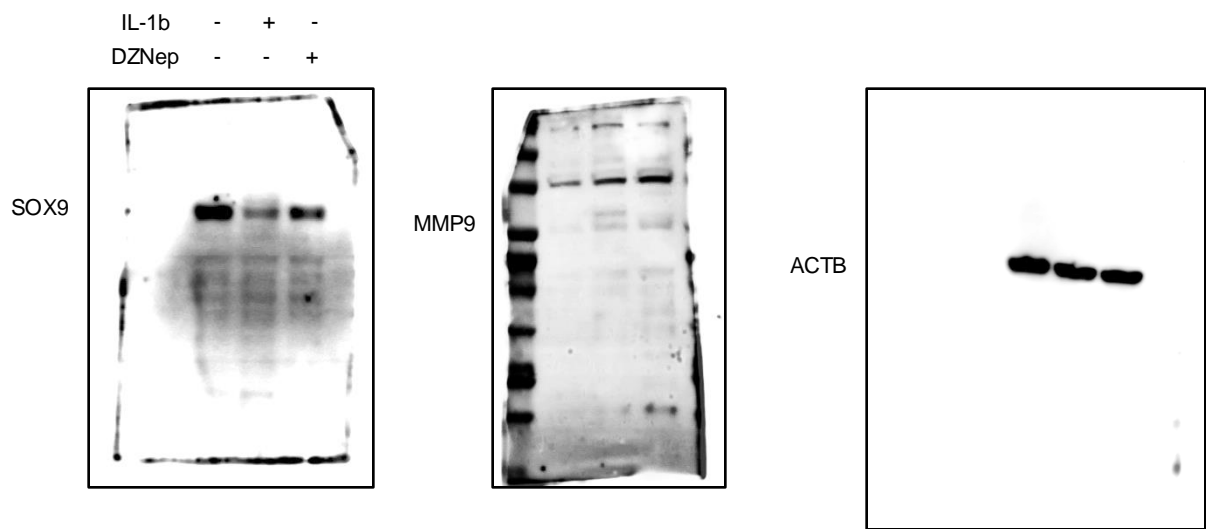

Figure 4F

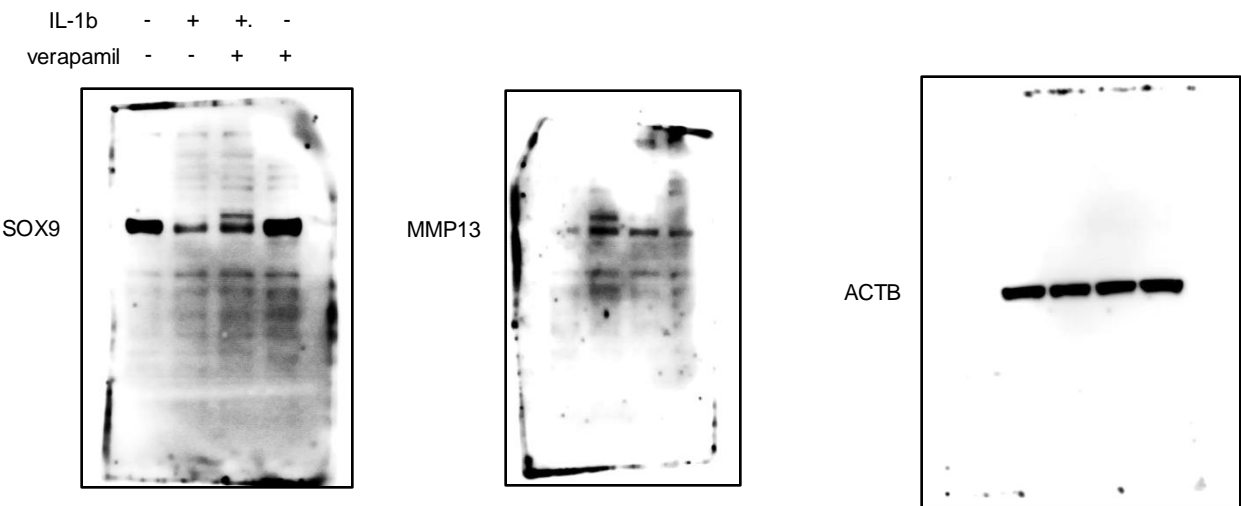

Supplement: Supplementary file 1 — Original wb images [file 41420_2025_2394_MOESM1_ESM.pdf]
